# Integrated phenotyping of root and shoot growth dynamics in maize reveals specific interaction patterns in inbreds and hybrids and in response to drought

**DOI:** 10.3389/fpls.2023.1233553

**Published:** 2023-09-01

**Authors:** Rongli Shi, Christiane Seiler, Dominic Knoch, Astrid Junker, Thomas Altmann

**Affiliations:** ^1^ Department of Molecular Genetics, Leibniz Institute of Plant Genetics and Crop Plant Research (IPK), Seeland, Germany; ^2^ Federal Research Centre for Cultivated Plants, Institute for Resistance Research and Stress Tolerance, Julius Kühn Institute (JKI), Quedlinburg, Germany

**Keywords:** maize, whole-plant phenotyping, root imaging, dynamic growth, hybrid, inbred, drought stress

## Abstract

In recent years, various automated methods for plant phenotyping addressing roots or shoots have been developed and corresponding platforms have been established to meet the diverse requirements of plant research and breeding. However, most platforms are only either able to phenotype shoots or roots of plants but not both simultaneously. This substantially limits the opportunities offered by a joint assessment of the growth and development dynamics of both organ systems, which are highly interdependent. In order to overcome these limitations, a root phenotyping installation was integrated into an existing automated non-invasive high-throughput shoot phenotyping platform. Thus, the amended platform is now capable of conducting high-throughput phenotyping at the whole-plant level, and it was used to assess the vegetative root and shoot growth dynamics of five maize inbred lines and four hybrids thereof, as well as the responses of five inbred lines to progressive drought stress. The results showed that hybrid vigour (heterosis) occurred simultaneously in roots and shoots and was detectable as early as 4 days after transplanting (4 DAT; i.e., 8 days after seed imbibition) for estimated plant height (EPH), total root length (TRL), and total root volume (TRV). On the other hand, growth dynamics responses to progressive drought were different in roots and shoots. While TRV was significantly reduced 10 days after the onset of the water deficit treatment, the estimated shoot biovolume was significantly reduced about 6 days later, and EPH showed a significant decrease even 2 days later (8 days later than TRV) compared with the control treatment. In contrast to TRV, TRL initially increased in the water deficit period and decreased much later (not earlier than 16 days after the start of the water deficit treatment) compared with the well-watered plants. This may indicate an initial response of the plants to water deficit by forming longer but thinner roots before growth was inhibited by the overall water deficit. The magnitude and the dynamics of the responses were genotype-dependent, as well as under the influence of the water consumption, which was related to plant size.

## Introduction

Plant phenotyping is essential for genetic mapping approaches as well as selecting elite lines from diverse germplasms in breeding. About half of the improvements in grain yield observed over the past seventy years have been attributed to improvements in cultivar genetics (Hauck et al., 2014). In maize, hybrids contribute greatly to increased yield and play an important role in breeding (Duvick, 2005; Li C. et al., 2022). With rapid technological advancements, modern plant phenotyping has been widely applied in plant research during recent decades (Costa et al., 2019). It is mainly performed using non-invasive methods to measure complex plant traits, such as growth and physiology dynamics over time (Walter et al., 2015; Costa et al., 2019). Plant phenotyping is considered a key tool for understanding plant growth and development and plant–environment interactions across different scales of resolution, from the cellular to the whole plant or plant stand level (Janni and Pieruschka, 2022). It supports fundamental plant research towards the elucidation of biological processes and mechanisms leading from genetic variation and interaction with the environment to the expression of important traits (Langstroff et al., 2022). Furthermore, it can speed up the characterization and improvement of agronomic traits enabling more sustainable agriculture as well as the development of new industrial products, such as biostimulants (De Diego and Spíchal, 2022).

Initially, most phenotypic analyses have focused on the aboveground parts of plants. Many desirable agronomic traits, hybrid performance-related traits, or stress adaption-related traits were assessed via imaging-based high-throughput shoot phenotyping (Junker et al., 2015; Neumann et al., 2015; Knoch et al., 2020). Adaptation to stress mainly involves morphological and physiological changes. These changes are controlled by molecular mechanisms that regulate the expressions of genes. Plant phenotyping helps identify genomic regions associated with trait and ultimately causal genes and genetic variants (Janni et al., 2019). For example, Wu et al. (2021) identified 1,529 QTL and 2,318 candidate genes related to drought responses by using a high-throughput system to study 368 maize genotypes, and further validated the functions of two candidate genes.

In recent years, the importance of roots has been increasingly appreciated by researchers. Roots display strong plasticity and are able to respond dynamically to local gradients of moisture and nutrients and shape their architecture to explore the heterogeneous soil according to the plant’s needs (López-Bucio et al., 2003; Hauck et al., 2015). Roots show plastic developmental responses to differences in nitrogen or other nutrients (Giehl et al., 2014; Jia et al., 2019) or water availability (Orman-Ligeza et al., 2018; Orosa-Puente et al., 2018; von Wangenheim et al., 2020) or to soil compaction (Pandey et al., 2021). The alteration of the root system architecture (RSA) by the *DEEPER ROOTING 1* (*DRO1*) gene, which was identified within a quantitative trait locus controlling root growth angle, improves drought avoidance in rice (Uga et al., 2013). Moreover, heterosis, the enhanced performance of hybrids compared to their inbred parents, is also manifested in roots (Hoecker et al., 2006) and Hauck et al. (2015) detected high variation and heterosis in traits of RSA and root complexity (the degree of branching) among 12 parental maize inbred lines and 66 F1 hybrids thereof using the excavated roots of field grown plants and a high-throughput imaging device. In order to support the assessment of root traits and thus to accelerate genetic analyses and investigations of mechanisms controlling root growth and development, as well as programs addressing the improvement of root traits important for plant performance, various root phenotyping facilities have been established. These include systems with artificial growth substrates such as agar or other media and platforms to monitor roots growing in soil (Iyer-Pascuzzi et al., 2010; Downie et al., 2012; Gioia et al., 2017; Shi et al., 2018), which offer different degrees of accessibility of the root in terms of visualizing the entire root system and in terms of the size to which the root system can grow.

At the whole plant level, the close interaction between the shoot and the root and their strong interdependence should be considered. When studying maize inbred lines released in different years, Ren and colleagues (2022) found that newly released inbred lines had steeper root angles. The results suggest that root traits were indirectly selected during modern breeding as breeders aimed at improving aboveground agronomic traits. By selecting shoot and root traits simultaneously and directly, it is possible to achieve genetic gain for the whole plant more quickly than selecting shoot or root traits alone (Tracy et al., 2020). However, to date, phenotyping studies have mostly focused on only the shoot or root system; there are quite limited platforms able to phenotype at the whole plant level (Nagel et al., 2012; Jeudy et al., 2016). With the GROWSCREEN-Rhizo, Nagel et al. (2012) presented a phenotyping system capable of automatic and simultaneous imaging of roots and shoots using soil-filled rhizotrons. However, the work focused mainly on characterizing root systems, and very few shoot phenotypic traits, such as the leaf area, were quantified. Shoot architecture-related traits or colour-related traits were not included. On the other hand, the relationship between shoot and root traits shows different patterns under various environmental conditions. Some researchers pointed out that altering the relationship among root and shoot traits is part of the strategies of plants to cope with drought (Lozano et al., 2020). Therefore, whole plant phenotyping covering both roots and shoots is required to gain a better understanding of the fundamental biological processes governing plant growth and development and ultimately plant performance.

The Leibniz Institute of Plant Genetics and Crop Plant Research (IPK), Gatersleben, operates and uses several automatic non-invasive high-throughput phenotyping platforms for different plant sizes in controlled-environment growth facilities, including a system suitable for large plants [described in Junker et al. (2015)]. The system facilitated the analysis of shoot phenotypes of diverse plant species such as maize (Muraya et al., 2017; Dodig et al., 2021) and rapeseed (Knoch et al., 2020). Increasing recognition of the importance of root system adaptation prompted us to extend our established shoot phenotyping platform for large plants with root phenotyping units based on a previously validated concept (Shi et al., 2018). In the following, we present two case studies illustrating the applicability of the platform: the vegetative root and shoot growth dynamics of 1) five maize inbred lines and four hybrids and 2) the assessment of the responses of five inbred lines to progressive drought stress. The upgraded phenotyping system will facilitate future research on different environmental cues and in different plant species by simultaneously analysing the dynamics of root and shoot growth.

## Materials and methods

### Plant materials and growth conditions

Four maize (Zea mays L.) hybrids: B73xUH007, N22xUH007, P148xUH007, and PHT77xUH007 and their parental inbred lines B73, N22, P148, PHT77, and UH007 were used for the first case study. Four of these inbred lines, B73, N22, P148, and PHT77, and one additional inbred line, S052, were used for the second case study. Each line had nine replicates (individual pots/plants). All the lines are part of the EPPN/EPPN2020 reference maize panel and were selected for the present study according to results of a previous investigation on the genetics of shoot growth (Muraya et al., 2017). The lines B73, N22, and PHT77 were initially provided by Alain Charcosset and Cyril Bauland, INRA(E) Mulon, France (see Rincent et al., 2014) and lines S052, P148, and UH007 were made available by Albrecht Melchinger, University of Hohenheim, Germany, and were propagated at IPK Gatersleben. Seeds of the hybrids B73xUH007, N22xUH007, P148xUH007, and PHT77xUH007 were supplied by Claude Welcker, INRA(E), LEPSE, Montpellier, France.

**Figure 1:**
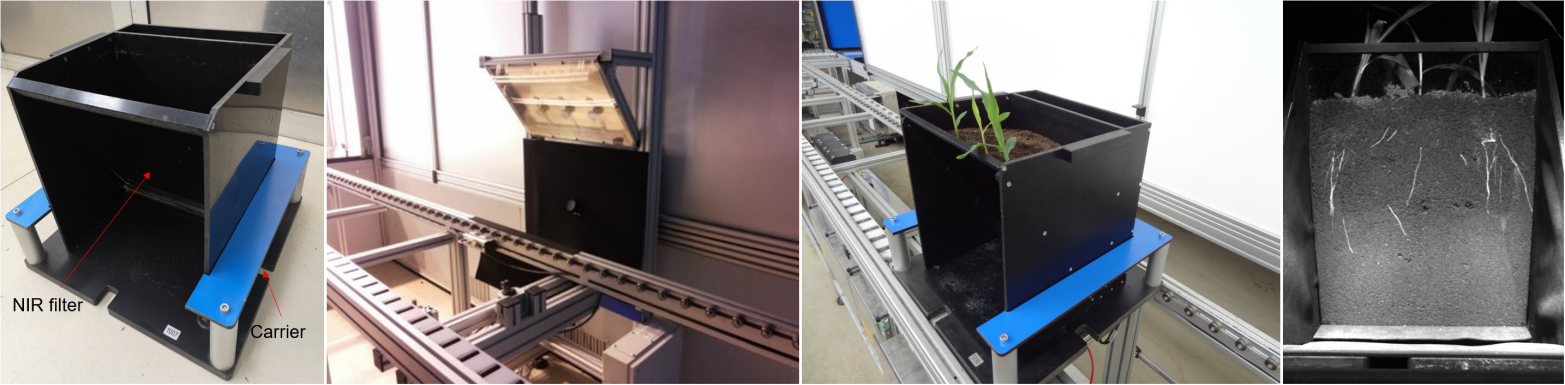
Custom-made ‘rhizo-pot’ for simultaneous root and shoot imaging. NIR filter: long pass near infrared filter plate allows only light above 750nm to pass through. The root images are taken by a NIR-sensitive camera directed at right angles to the NIR filter plate upon illumination with NIR (850 nm) emitting diodes.

Seeds were germinated on wet filter paper and, after 4 days, seedlings were transplanted into the custom-made ‘rhizo-pots’, one plant per pot. The special rhizo-pot was designed based on the root phenotyping concept, which was validated in our previous work (Shi et al., 2018) and the prototype was pre-tested in the system. The bottom and three sides of the box are made of black PVC in order to prevent the roots from being exposed to light. The front side is tilted 30 degrees allowing the roots to be visualized effectively. A NIR filter which allows the spectrum above 750 nm to pass through is inserted at this side. The plants were transplanted close to the NIR filter, and the root images were taken only from this side. The size of the box is 35 x 25 x 40 cm (LxBxH; **Figure 1**). The boxes were filled with about 7 kg of black peat soil (Graberde, Plantaflor, Germany). The top surface of the boxes were covered with a black mesh to improve image quality and reduce water evaporation. All the rhizo-pots were placed in carriers and entered the conveyor belt-based automated plant phenotyping system. The system is located at the IPK in a climatized greenhouse and plants were grown under controlled long-day conditions with 25/20 °C and 16/8 h day/night, as descripted by Junker et al. (2015).

All the hybrids and inbred lines used for the first case study were grown only under well-watered conditions (60% field capacity (FC)). The FC was determined on a gravimetric basis as described by Junker et al. (2015). Briefly, soil water content corresponding to 100% field capacity was determined by weighing soil-filled pots (0.3L) after watering to full saturation (100% FC) and weighing them after drying the soil completely (20 days at 70 °C). The weight corresponding to 60% or 35% field capacity was then calculated accordingly. For the second case study, the five inbred lines were grown under well-watered (WW) and drought (D) conditions within the same experiment. The plants of the well-watered inbred lines B73, PHT77, P148, and N22 were the same in both studies. Water supply was maintained by automated weighing and watering towards target weight and was stopped at 13 days after transplanting (DAT) to induce drought stress and was kept to 35% FC. Five soil moisture sensors (Decagon 5TE, UMS, Germany) were inserted in the soil at a depth of 10 cm for each genotype under different treatment to record the water content in the pot. All plants were fertilized with Hakaphos Blau (Compo Expert, Germany) 150 ml/pot at 11 DAT. The hybrids and WW plants were fertilized two times more at 21 DAT and 27 DAT.

In order to further evaluate the newly integrated root phenotyping system, additional plants of two inbred lines B73 and N22 (each with seven replicates) were cultivated simultaneously with the plants of the two case studies and sampled at 19 DAT. These two lines have contrasting root biomass as shown in our previous work (Shi et al., 2018): B73 has a relatively large root biomass while N22 has a small root biomass. At the end of the experiment, roots of B73 and N22 were dug out, washed, and placed in a 28 x 40 cm transparent tray filled with distilled water. The roots were scanned at 400 dpi on an Epson Expression 10000 XL scanner (Seiko Epson).

The V stage (the number of visible leaf collars) and leaf number were counted manually after 13 DAT on a weekly basis. At 40 DAT, shoots were cut from the base and the fresh and dry weight (oven-dry at 70˚C for one week) were determined.

### Imaging acquisition and analysis

After transplanting, visible light (RGB) and static fluorescence (FLUOR) top view and side view images of shoots, as well as near infra-red (NIR) images of roots, were taken on a daily basis from 4 to 40 DAT (4 to 39 DAT for roots). Due to technical failures, time points were missing at 9 (only for root), 30, and 35 to 39 DAT. Root images were taken by a CMO Mono sensor with 12 Mp resolution (UI-5200SE-M-GL Rev.4, iDS, Germany) which was integrated into the system. During imaging, an LED (LZ4-00R408 peak: 850 nm, range 835-875 nm) panel was used for NIR illumination.

To extract image-based shoot traits, the Integrated Analysis Platform (IAP) software (version 2.0; Klukas et al., 2014) was used with a customized analysis pipeline. From the very elaborate output of the image analysis with 445 phenotypic traits, the shoot traits ‘estimated shoot biovolume (px^3^)’ (ESV; biomass-related), ‘projected leaf area (px^2^)’ (PLA; biomass-related), ‘estimated plant height (px)’ (EPH; architecture-related), and the ‘brown to green ratio’ (colour-related) were selected and presented. They are based on visible light and from a combined view, top view, side view, and side view, respectively.

The scanned roots from B73 and N22 were analysed by WinRhizo Pro ver. 2013c (Regent Instruments). Root images derived from the phenotyping facility were pre-processed and analysed by the semi-automated Root Image Analysis (saRIA) software (Narisetti et al., 2019). It supports efficient image segmentation on soil-root images, while user input for selecting the best combination of algorithmic parameters is required. Noisy regions could be manually removed as well (Narisetti et al., 2019). The root traits ‘total root length (px)’ (TRL), ‘total root surface area (px^2^)’ (TRSA), ‘total root volume (px^3^)’ (TRV), and ‘average root diameter (px)’ (RD) were used for further analysis. The NIR root images from B73 and N22 at 19 DAT, which were used for validation, were additionally analysed by the SmartRoot software (Lobet et al., 2011). The root trait values extracted from images acquired after 29 DAT were not considered for statistical analysis as they were regarded as unreliable due to the increasing density of the root system and the progressive merging and overlapping of roots. Nevertheless, values derived from images taken at two time points, 34 and 39 DAT, were included in the figures, but only for illustrative purposes (shaded grey in the figures).

For the first case study, mid-parent heterosis (MPH in %) was calculated as the difference between hybrid performance (F1) and the mean value of the two parents [MP=(P1+P2)/2] for each trait at all time points as Eq.1.


(Eq. 1) $$\text{MPH}=\frac{\left(\text{F}1-\text{MP}\right)}{\text{MP}}\times 100$$

To evaluate the drought tolerance of the lines, the biomass ratio was assessed by comparing the biomass under drought (D) with the biomass under well-watered (WW) conditions. The calculation was done as follows (Eq.2-Eq.5; Fischer and Maurer, 1978; Correia et al., 2022). Shoot DW ratio was calculated based on the data at the end of the experiment, while the estimated shoot biovolume (ESV), total root length (TRL), and total root volume (TRV) were derived from the daily acquired images. Mean values were calculated for each day of the growth period after drought was imposed at 13 DAT.


(Eq. 2) $$\text{Shoot DW ratio}=\frac{\text{shoot }{\text{DW}}_{\text{D}}}{\text{shoot }{\text{DW}}_{\text{WW}}}$$

(Eq. 3) $$\text{ESV ratio}=\frac{{\text{ESV}}_{\text{D}}}{{\text{ESV}}_{\text{WW}}}$$

(Eq. 4) $$\text{TRL ratio}=\frac{{\text{TRL}}_{\text{D}}}{{\text{TRL}}_{\text{WW}}}$$

(Eq. 5) $$\text{TRV ratio}=\frac{{\text{TRV}}_{\text{D}}}{{\text{TRV}}_{\text{WW}}}$$

### Statistical analysis

The manually measured traits were analysed by an analysis of variance (ANOVA) or t-test using GENSTAT software ver. 16.0. Correlations between traits were analysed using the Pearson product moment correlation. The data visualization for phenotyping data was performed using the R software (R Core Team, 2019). Significant differences between the treatments for each day and trait were determined by one-way ANOVA at a significance level of 0.05.

## Results

### Validation of the root phenotyping implementation

Two genotypes contrasting in root biomass, B73 and N22, each analysed with seven replicates, were sampled at 19 days after transplanting (DAT) and used to validate the root phenotyping setup in our phenotyping platform. To this end, roots were dug out manually, washed, scanned, and root morphological traits were analysed using the WinRhizo (SC) software to generate ground truth data. The high-throughput phenotyping images were processed with ‘SmartRoot’ (SR) and ‘Semi-automated Root Image Analysis’ (saRIA).

The correlations between root dry weight (RDW) and root traits obtained by different software tools are shown in **Table 1**. There were high positive correlations among all three root traits: total root length (TRL), total root surface area (TRSA), and total root volume (TRV). The TRV obtained by scanning the root system displayed the highest correlation with RDW (r = 0.99). TRV reflected RDW better than TRL, regardless which software was used. Notably, the traits obtained by the saRIA software showed higher correlation with RDW than the traits obtained by the SmartRoot software which required manually tracing the roots. Therefore, the saRIA software was used to analyse root traits in the two case studies performed.

**Table 1:** Correlations between RDW (root dry weight), root traits TRL (total root length), TRSA (total root surface area), and total root volume (TRV) analysed by different software (n=14).

	SC-TRL	SC-TRSA	SC-TRV	SR-TRL	SR-TRSA	SR-TRV	saRIA-TRL	saRIA-TRSA	saRIA-TRV
RDW	0.967	0.985	0.99	0.915	0.941	0.959	0.939	0.953	0.963
SC-TRL	–	0.993	0.974	0.922	0.933	0.937	0.942	0.950	0.947
SC-TRSA		–	0.994	0.932	0.949	0.958	0.947	0.958	0.961
SC-TRV			–	0.929	0.952	0.965	0.939	0.954	0.962
SR-TRL				–	0.992	0.966	0.944	0.955	0.948
SR-TRSA					–	0.990	0.956	0.966	0.968
SR-TRV						–	0.963	0.972	0.982
saRIA-TRL							–	0.993	0.987
saRIA-TRSA								–	0.995
saRIA-TRV									–

SC (WinRhizo), SR (SmartRoot), and saRIA (Semi-automated Root Image Analysis) refer to the used software. The values denote correlation coefficient (R) between the traits analysed by Pearson correlation. The correlations were all statistically highly significant at p< 0.001.

Dynamic shoot and root growth of hybrid and inbred maize genotypes.

### Manually measured shoot traits

**Figure 2:**
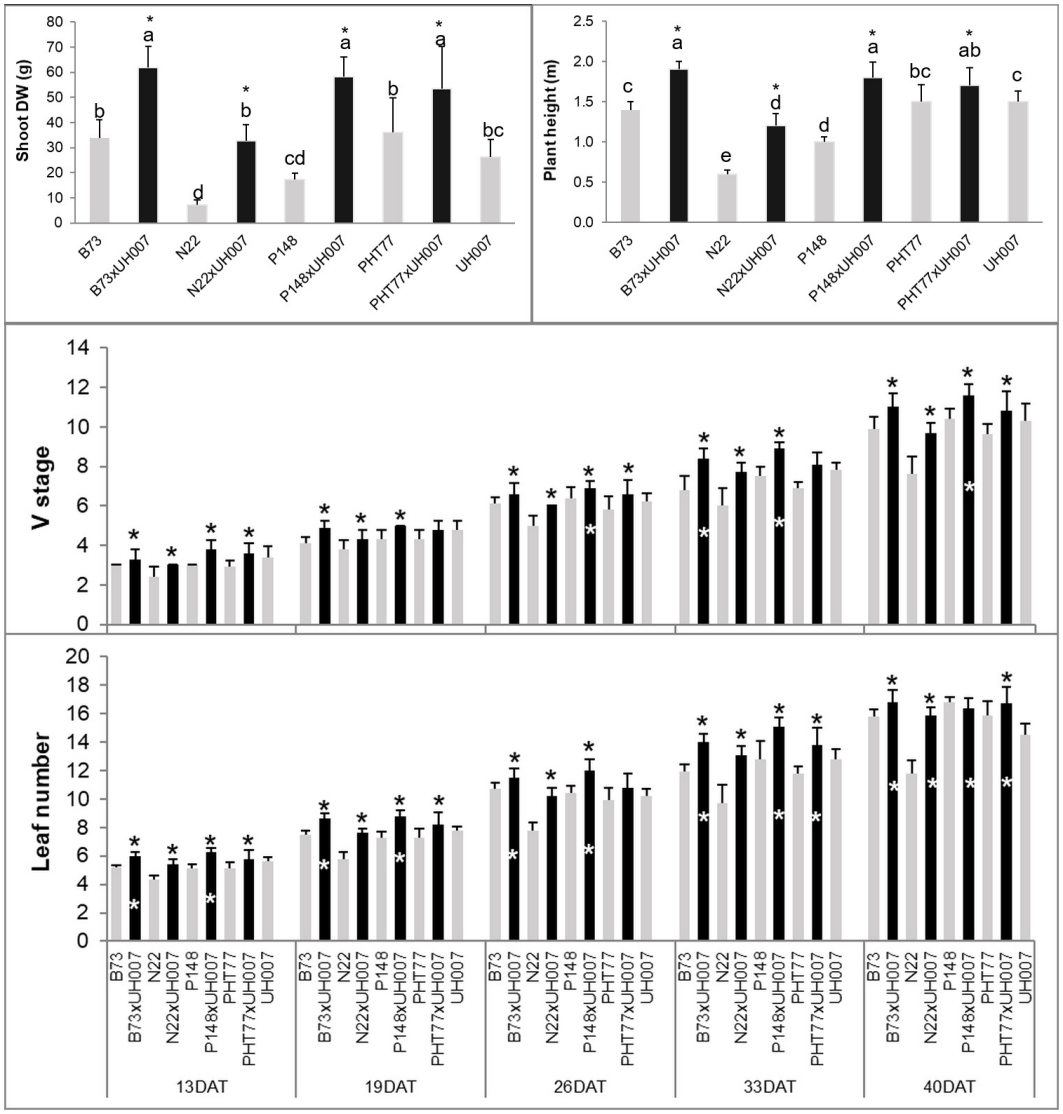
Manually measured plant height and shoot dry weight (DW) at the end of the experiment, V stage, and leaf number over time of hybrid and inbred lines. DAT: days after transplanting. Bars indicate means ± SD (n = 9). A black * indicates significant differences between the hybrid and the female parent inbred lines, while a white * indicates significant differences between the hybrid and the male parent inbred line UH007 compared by t-test (p<0.05). Different letters indicate statistically significant differences among all the lines (p<0.05) determined by ANOVA and Tukey’s HSD test.

At the end of the experiment, most hybrids (B73xUH007, N22xUH007, P148xUH007, and PHT77xUH007) had a significantly higher plant height (PH) and shoot dry weight (DW) compared with the corresponding female parental inbred lines (B73, N22, P148, and PHT77) or the male tester UH007 (**Figure 2** and **Supplementary Figure 1**). The only exception was the DW difference between N22xUH007 and UH007. The manually measured traits V stage and leaf number, both representing the development of the maize plants, increased over time. At 13 DAT, the hybrids were further developed, displaying a higher V stage and leaf number than the female inbred lines. Significant differences in V stage between UH007 and the hybrids were detectable starting at 26 DAT (P148xUH007). For leaf number, B73xUH007 and P148xUH007 had significantly more leaves than UH007 at 13 DAT and all the hybrids had a higher leaf number than UH007 at 40 DAT, similar to the other female inbred lines. Among the inbred lines, N22 displayed significantly lower shoot DW and a lower plant height compared with B73, PHT77, and UH007. During the whole growth period, N22 developed fewer leaves than the other inbred lines, which could be observed as early as 13 DAT.

### Image-derived shoot and root phenotypic traits

**Figure 3:**
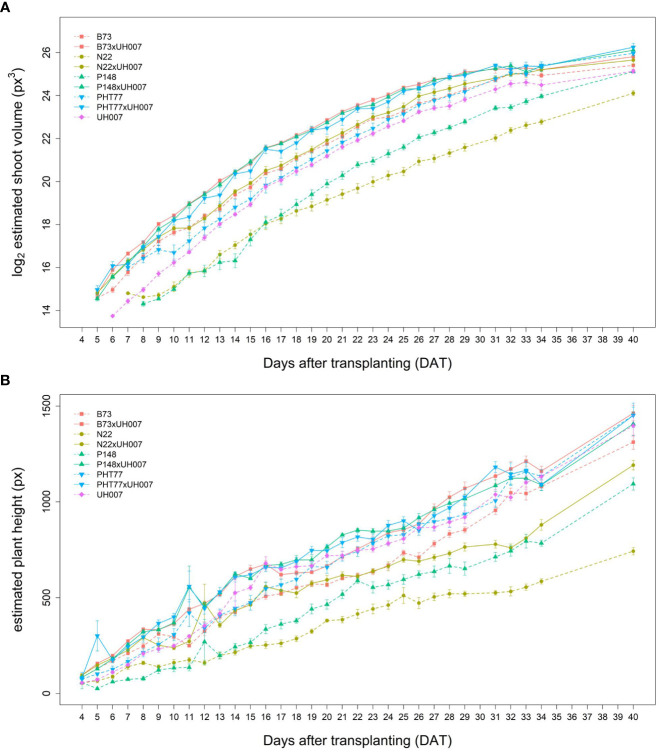
Estimated shoot biovolume (ESV) **(A)** and estimated plant height (EPH) **(B)** derived from the images of hybrid and inbred maize plants over time. Data are shown as means of nine replicates and the error bars denote ± SE.

Similar tendencies of the manually measured traits were also observed for the image-derived shoot traits. Estimated shoot biovolume (ESV), estimated plant height (EPH), and projected leaf area (PLA) from 4 to 40 DAT are shown in **Figures 3A, B** and **Supplementary Figure 2**, respectively. Due to a technical failure at 30 DAT, no imaging and watering was performed on this particular day. There were also no images between 35-39 DAT, but watering was carried out every second day during this period. Both hybrid and inbred lines showed continuous ESV, PLA, and PH increases over time. There was more variation at the later stages, most likely caused by losing some old leaves and/or greater overlap of leaves. As mentioned by Scharr et al. (2016), with the growth of plants, leaves tend to overlap which could result in less accurate estimates of the leaf area due to partial occlusion.

**Figure 4:**
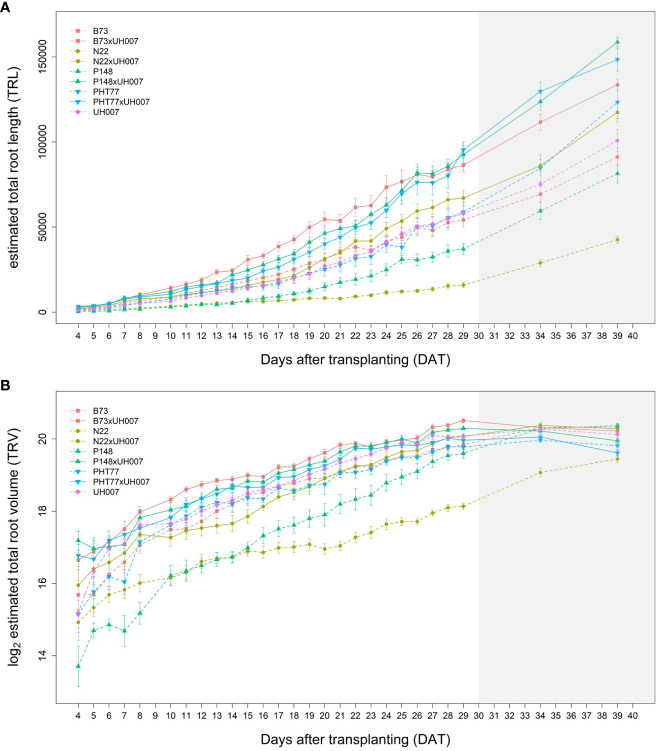
Estimated total root length (TRL) **(A)** and total root volume (TRV) **(B)** of hybrid and inbred maize plants over time extracted from NIR-images by saRIA (semi-automated Root Image Analysis) software. Data are shown as means of nine replicates and the error bars denote ± SE. The grey area marks time points with data of low reliability due to the increasing density of roots and their progressive merging and overlapping. Values derived from images taken at 34 and 39 DAT are included only for illustration.

The traits TRL, TRV, and TRSA were extracted from the root images acquired by the NIR camera and their changes over time (from 4-29 DAT) were analysed (**Figures 4A, B** and **Supplementary Figure 3**, respectively). In concordance with shoot growth, the hybrid lines displayed higher TRL values compared to the inbred lines almost throughout the whole growth period. This tendency could be observed already during the early growth phase at 4 DAT (**Table 2**). At 29 DAT, the hybrids B73xUH007, N22xUH007, P148xUH007, and PHT77xUH007 had a 1.6, 4.2, 2.5, and 1.6 times greater TRL than their respective parental inbred lines B73, N22, P148, and PHT77. Among the inbred lines, B73, PHT77, and UH007 had similar TRL, and they were substantially higher compared with N22 (**Figure 4A**). Roots of N22 were not only shorter, but also displayed a limited volume, which reflected a smaller root system. TRV showed a similar tendency as TRL regarding the growth pattern of hybrids and inbred lines, except that UH007 had the highest TRV among all inbred lines, which was even higher than the hybrid N22xUH007. This mainly resulted from the bigger RD, as show in **Supplementary Figure 3**.

**Table 2:** Statistical analysis by using ANOVA (analysis of variance) between hybrid and inbred lines, as well as well-watered (WW) and drought-treated (D) maize plants.

	Hybrid vs. Inbred				WW vs. D			
DAT	ESV	EPH	TRL	TRV	ESV	EPH	TRL	TRV
4	– ^a^	*	***	***	NS ^b^	NS	NS	NS
5	–	*	***	***	NS	NS	NS	NS
6	***	***	***	***	NS	NS	NS	NS
7	***	***	***	***	NS	NS	NS	NS
8	***	***	***	***	NS	NS	NS	NS
9	***	***	***	***	NS	NS	NS	NS
10	***	**	***	***	NS	NS	NS	NS
11	***	**	***	***	NS	NS	NS	NS
12	***	*	***	***	NS	NS	NS	NS
13	***	***	***	***	NS	NS	NS	NS
14	***	***	***	***	NS	NS	NS	NS
15	***	***	***	***	NS	NS	NS	NS
16	***	**	***	***	NS	NS	NS	NS
17	***	***	***	***	NS	NS	NS	NS
18	***	***	***	***	NS	NS	NS	NS
19	***	***	***	***	NS	NS	NS	NS
20	***	***	***	***	NS	NS	NS	NS
21	***	***	***	***	NS	NS	NS	NS
22	***	***	***	***	NS	NS	NS	NS
23	***	***	***	***	NS	NS	NS	NS
24	***	***	***	***	NS	NS	NS	*
25	***	***	***	***	NS	NS	NS	*
26	***	***	***	***	NS	NS	NS	**
27	***	***	***	***	NS	NS	NS	***
28	***	***	***	***	NS	NS	NS	***
29	***	***	***	***	*	NS	NS	***
31	***	***			*	NS		
32	***	**			***	*		
33	***	**			**	*		
34	***	*			***	**		
35	–	–			–	–		
39	–	–			–	–		
40	***	**			***	***		

*indicates p< 0.5, **p<0.1, and ***p< 0.01. Analysis results of values derived from root images taken from 30 to 39 DAT are not shown due to the increasing density of roots and their progressive merging and overlapping.

^a^denotes no data, ^b^denotes no significant difference.

ESV, estimated shoot biovolume; EPH, estimated plant height; TRL, total root length; TRV, total root volume; NS-no significant difference.

**Figure 5:**
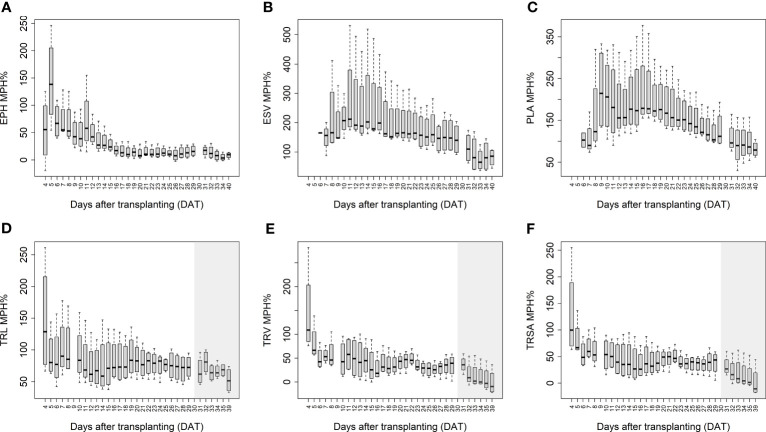
Mean mid-parent heterosis (MPH) for estimated plant height (EPH) **(A)**, estimated shoot biovolume (ESV) **(B)**, projected leaf area (PLA) **(C)**, total root length (TRL) **(D)**, total root volume (TRV) **(E)**, and total root surface area (TRSA) **(F)** across hybrids by using image-derived traits. The grey area marks time points with data of low reliability due to the increasing density of roots and their progressive merging and overlapping. Values derived from images taken at 34 and 39 DAT are included only for illustration.

The mean mid-parent heterosis (MPH) for the hybrids was calculated based on the imaging-derived traits of both shoots and roots (**Figure 5**). Generally, the MPH of all traits varied dynamically over time with higher MPH values at the early stage. TRL, TRV, and TRSA especially displayed high MPH at 4 DAT, though with quite high variation. The highest MPH for EPH was found at 5 DAT which reached 144%. The MPH of ESV and PLA showed the highest value several days later, in the period of 9 to 10 DAT, and both reached more than 200%. It seems that heterosis was manifested earlier in roots than in shoots, while the degree of heterosis was lower in roots compared to shoots. The range of MPH for TRL and TRV (4-29 DAT) was 70-146%, and 28-144%, while MPH for ESV and PLA (4-40 DAT) it ranged between 73-217% and 81-224%, respectively.

### Shoot and root growth dynamics under drought stress

**Figure 6:**
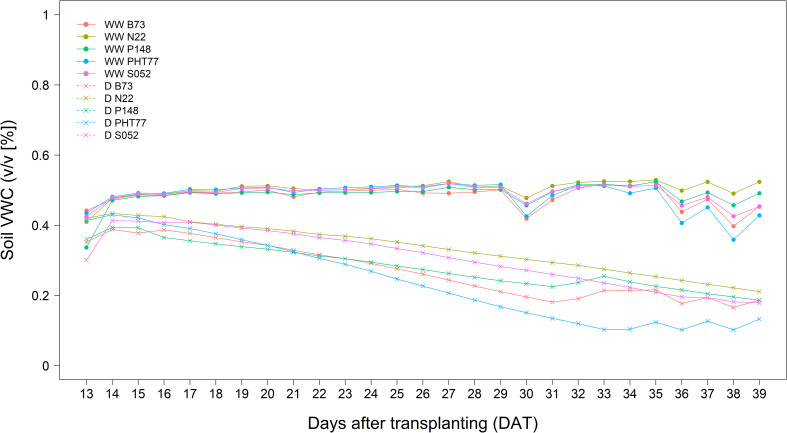
Soil volumetric water content (VWC (v/v [%])) determined by a soil sensor in well-watered (WW) and drought-treated (D) pots. The values denote the mean of five replicates of each line.

The inbred lines B73, N22, P148, PHT77, and S052 were evaluated for their phenotypic response to drought. Drought stress was induced by stopping the water supply starting from 13 DAT. Due to the large soil volume in the rhizo-pot, the soil moisture level dropped down progressively as shown either by soil VWC measured with soil sensors (**Figure 6**) or by the calculated field capacity (FC; **Supplementary Figure 4**). Both methods showed the same tendency that only at a late stage, after 33 DAT, did the FC of the soil in the pots of B73 and PHT77 decrease to 35%. For P148 and S052, the FC dropped to 40% during the last two days, while the pots of N22 still had about 45% FC at the end of the experiment (**Supplementary Figure 4**).

### Manually measured shoot traits

**Figure 7:**
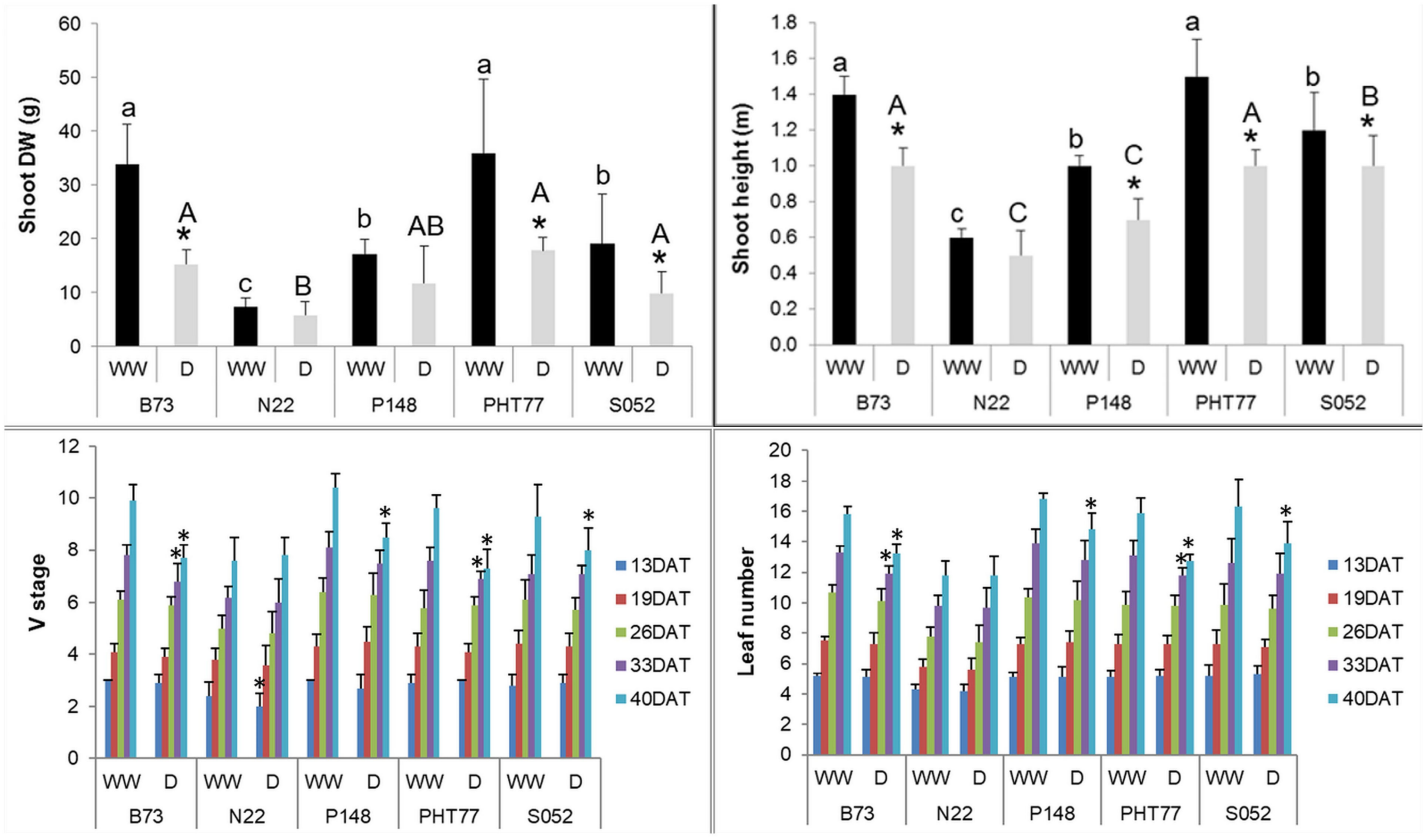
Manually measured shoot height and shoot dry weight (DW) at the end of the experiment, V stage, and leaf number over time. DAT: days after transplanting. Bars indicate means ± SD (n = 9). * indicates significant differences between well-watered (WW) and drought-treated (D) plants (p<0.05). Different letters indicate statistically significant differences among the genotypes under well-watered (small letters) or drought (capital letters) condition (p<0.05).

Drought stress significantly decreased PH and shoot DW of B73, PHT77, and S052. Although P148 displayed a decreased PH, the DW did not differ significantly between well-watered and drought-stressed plants. Both PH and shoot DW were unaffected by the drought treatment in N22, likely due to a lower stress intensity with a relatively high FC (higher than the intended 35%) even at the late growth stages (**Figure 7**). The V stage and leaf number of B73 and PHT77 were affected by the drought from 33 DAT, while only at 40 DAT, a substantial effect was observed on P148 and S052. Similar to PH and shoot DW results, the V stage and leaf number did not differ between the two treatments in N22 (**Figure 7**).

### Image-derived shoot and root phenotypic traits

**Figure 8:**
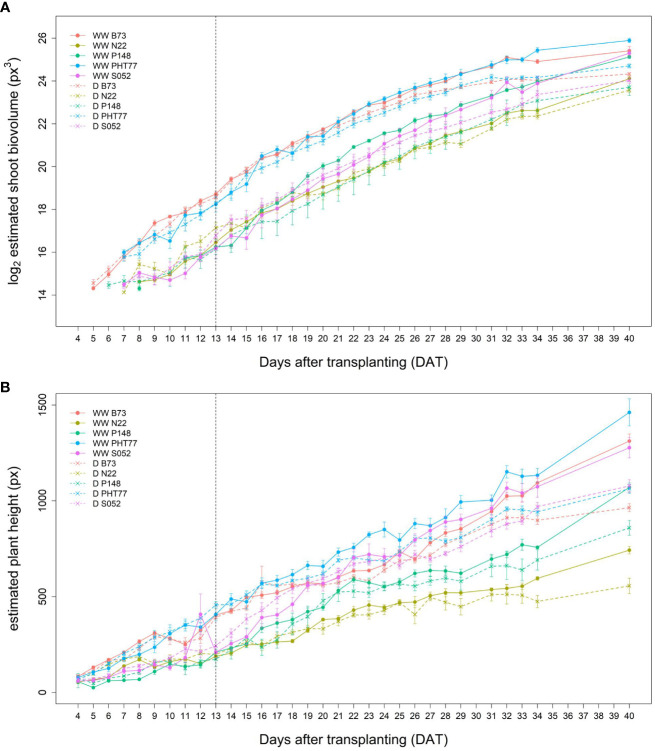
Estimated shoot biovolume (ESV) **(A)** and estimated plant height (EPH) **(B)** derived from the images of well-watered (WW) and drought-treated (D) plants over time. Data are shown as means of nine replications and the error bars denote ± SE. The vertical dashed line denotes the starting time of imposing drought stress at 13 DAT.

It took several days for plants to show detectable phenotypic changes after the drought stress was imposed. Starting from 29 DAT, a significant difference in ESV appeared between well-watered and drought-treated plants (**Table 2**). Drought tended to reduce PH for all the tested inbred lines over time, although significant differences were not detectable until 32 DAT. The ESV and EPH at 40 DAT were 2.3 and 1.35 times higher, respectively, under well-watered conditions compared with drought treatment (**Figures 8A, B**). B73 and PHT77 were most severely affected by the drought stress. Their ESV and PLA decreased about ten days after drought imposition, while there was no significant difference detectable in N22 at that time (**Figure 8A**; **Supplementary Figure 5**). Most colour-related shoot traits, such as the brown to green ratio (**Supplementary Figure 6**), showed no obvious changes due to the drought stress.

**Figure 9:**
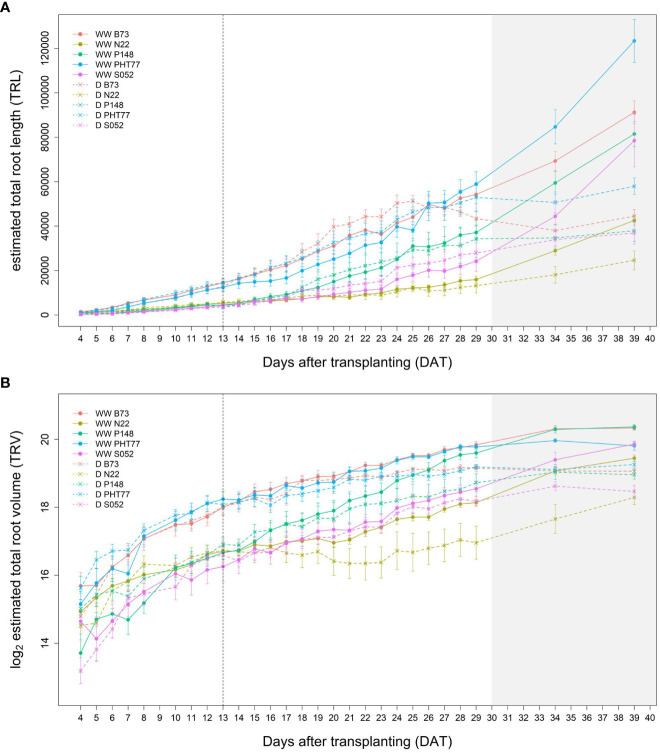
Estimated total root length (TRL) **(A)** and total root volume (TRV) **(B)** of well-watered (WW) and drought-treated (D) plants over time extracted from the NIR-images by saRIA (semi-automated Root Image Analysis) software. Data are shown as means of nine replications and the error bars denote ± SE. The vertical dashed line denotes the starting time of imposing drought stress at 13 DAT. The grey area marks time points with data of low reliability due to the increasing density of roots and their progressive merging and overlapping (particularly in the WW plants). Values derived from images taken at 34 and 39 DAT are included only for illustration.

Root growth of all tested lines was substantially affected by the drought stress (**Figures 9A, B** and **Supplementary Figure 7**). Water deficit significantly decreased TRV compared to well-watered plants, and this was evident from 24 DAT, about 10 days after the watering was stopped (**Figure 9B** and **Table 2**). TRL showed a trend toward higher TRL in the drought-treated plants, although the differences between 18 and 23 DAT were not significant (**Figure 9A**). Subsequently, the TRL of most drought-treated plants stagnated or decreased over time and was on average 10% lower than that of the well-watered plants at 29 DAT. RD of all the lines were decreased under drought stress (**Supplementary Figure 7**).

**Figure 10:**
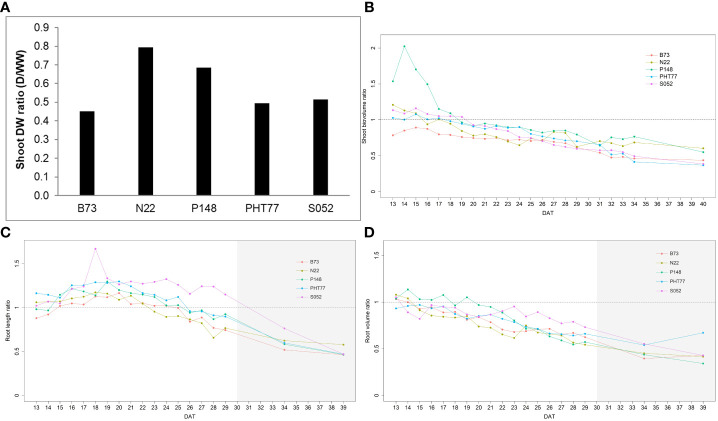
Shoot DW ratio (shoot DW under drought/shoot DW under well-watered) of the five inbred lines at the end of the experiment at 40 DAT **(A)**. Estimated shoot biovolume ratio (ESV ratio) over time after drought imposition at 13 DAT **(B)**. Estimated total root length (TRL) and total root volume ratio (TRV) over time **(C, D)**. The mean values of nine replicates under each treatment were used to calculate the ratio. The dashed line denotes 1 which means the trait had the same value in the drought and the well-watered (WW) conditions. DAT: days after transplanting. The grey area marks time points with data of low reliability due to the increasing density of roots and their progressive merging and overlapping (particularly in the WW plants). Values derived from images taken at 34 and 39 DAT are included only for illustration.

Drought stress reduced the shoot DW for all tested inbred lines (**Figure 10A**). Among the lines, B73 was most severely affected by the drought treatment and gained only 45.3% of shoot biomass of the corresponding well-watered plants, while N22 was least affected (79.5%). After one week of drought stress, the ESV ratio between drought and well-watered plants dropped below a value of 1 for all lines. The value continuously decreased over time and reached on average about 0.5 at 40 DAT (**Figure 10B**). There was variation in the degree of reduction in the five inbred lines. Starting from 32 DAT, the observed tendency became stable. Consistent with the end-point results at 40 DAT, the ESV ratios for B73, PHT77, and S052 were lower than for P148 and N22, indicating a stronger effect on those lines. Root growth was affected by the water deficit as well, as shown in **Figures 10C, D**. The TRV ratio decreased over time, and it appears there were genotypic differences between the lines. After ten days of water deficit, the TRV of S052 was much less reduced than the TRV of the other lines (**Figure 10C**). The ratio of TRL showed a different pattern. The ratio was above 1 for all lines during more than half of the drought stress period from 15 to 25 DAT. This increased root length (ratio >1) lasted for about ten days, and then decreased to an average value of 0.83 at 29 DAT (except for S052). Notably, for S052 the TRL increased for a period of 14 days (15 to 29 DAT), while for N22 it only increased for 7 days (15 to 22 DAT; **Figure 10D**).

## Discussion

Although diverse high-throughput phenotyping facilities have been developed in recent years, platforms capable of simultaneously assessing both the root and the shoot of plants are rare. It has been reported that the growth and structure of the belowground and aboveground parts of plants affect each other and, hitherto, this relationship has been particularly investigated in trees by ecologists (Parsons et al., 1994; Wang et al., 2023). However, also in annual plants like maize, effects of aerial conditions such as solar radiation can affect both shoot and root growth and can cause shifts in the root/shoot ratio (Guo et al., 2021). As reported by Su et al. (2019), the growth of plant shoots is closely associated with the size of the root system. They found nitrogen efficient maize hybrids had deeper and bigger roots and higher grain yield than nitrogen inefficient lines at low nitrogen application rates, although the mechanism of the interaction between shoots and roots is still unclear. Therefore, further studies with suitable phenotyping facilities are necessary to examine root and shoot traits in a single framework.

### Integrated root phenotyping

The non-invasive high-throughput shoot phenotyping platform at IPK has been utilized in many research studies for diverse models and crop plants (Junker et al., 2015; Muraya et al., 2017; Knoch et al., 2020; Dodig et al., 2021). As realized by many researchers, crop species with optimised root systems are essential for future food security and key to improving agricultural productivity and sustainability (Li A. et al., 2022). In order to enhance our understanding of the root system, in particularly the dynamics of root growth and development, a root phenotyping concept was developed (Shi et al., 2018) and root phenotyping units were established and integrated in the existing phenotyping platform for large plants. In this work, we present the upgraded facility and the use of the root phenotyping units to evaluate jointly shoot and root growth in two case studies.

The high positive correlations between root DW and image-derived traits such as TRL and TRV indicate that our root phenotyping facility is well suited to monitor root growth (**Table 1**). The root analysis software saRIA (Narisetti et al., 2019) was utilized to analyse the root images obtained by the automated high-throughput phenotyping system. This upgraded system now enables simultaneous non-invasive analysis of root and shoot traits of the same plants in a particular phenotyping experiment.

### Shoot and root growth dynamics in maize hybrid and inbred lines

Hybrids often display superior phenotypes due to their vigorous nature (van Dijk et al., 2016), a phenomenon widely known as heterosis. The superior performance of F1 hybrids compared to their parental inbreds has been known for decades, although the underlying genetic and regulatory mechanisms remain largely unclear (Paschold et al., 2010). Heterosis studies rely on morphological and physiological analyses of inbred lines and corresponding hybrids. So far, most related research has focused either on shoot or root traits independently using separate approaches.

In the present study, the dynamics of vegetative shoot and root growth were investigated in a selection of maize hybrid and inbred lines. Growth-related image-derived shoot and root traits were analysed during the early vegetative growth phase, from 4 to 40 DAT. The phenotyping results indicated an early establishment of heterosis in the tested hybrids. EPH, TRL, and TRV were higher in hybrids than in their corresponding parental inbred lines as early as 4 DAT, the first day when shoot and root images were taken (**Table 2**). At the end of the growth period, EPH and shoot DW in hybrids were on average 20% and 55% higher than in their parental inbred lines, respectively. These findings are consistent with the results of previous studies, which showed the superiority of hybrids (Hauck et al., 2014; Zhou et al., 2018). The range of MPH for ESV (73-217%) and PLA (81-224%) was similar to that reported by Tollenaar et al. (2004). In their study, they reported MPH values from 138% to 214% for dry matter accumulation and 92% to 204% for leaf area at the 14-leaf stage, respectively. Thanks to the better performance with superior biomass and higher seed yield, hybrid maize varieties have been predominantly grown worldwide since the 1960s (Li C. et al., 2022). Further increasing yield potential and yield stability through heterosis remains a major goal of maize breeding (Duvick, 2005; Li C. et al., 2022). The advances in high-throughput phenotyping facilities will assist and fasten this process by supporting the gain of fundamental genetic and mechanistic knowledge.

In addition to the aboveground parts, heterosis is also observable in the belowground organs of plants. In accordance with Hoecker et al. (2006), the primary root length, number of seminal roots, and the lateral root density can display substantial heterosis. They demonstrated that heterosis manifests in the very early stages of root development a few days after germination. We observed in our study that hybrids displayed high MPH values as early as 4 DAT (8 days after germination), with on average 67% and 70% higher TRL and TRV values than the inbred lines, respectively (**Figure 5**). Compared to conventional methods of quantifying trait expression at one particular time point (usually using destructive techniques), heterosis in shoots and roots can be assessed here in a dynamic manner by a non-invasive, fast, and high-throughput procedure. We observed that heterosis occurred earlier in roots than in shoots and decreased over time in both organs.

The ability to compare heterosis for both shoot and root simultaneously, even under various conditions, will help researchers to further explore the developmental and physiological mechanisms associated with heterosis and to jointly study the genetic basis of heterosis for both organ systems, which are highly interdependent.

### Shoot and root growth dynamics in maize in response to drought

Drought stress is one of the most serious adverse environmental factors limiting crop productivity and a major threat to world food security (Boyer et al., 2013; Berdugo et al., 2020). Due to global climate change, the frequency and duration of drought periods will most likely increase (Trenberth et al., 2014). Therefore, it is of the utmost importance to understand how plants respond and adapt to water deficit in order to support solutions to this problem and enhance the sustainability of agricultural production.

In the present study, drought stress was induced at 13 DAT and drought symptoms such as leaf wilting and rolling could be observed in B73 and PHT77 at the growth stages V6-V10. The effect of drought was reflected by decreases in PLA and ESV, as well as in the final shoot DW. Plants generally decline the number and area of leaves in response to drought stress. This was confirmed by our manually measured parameters and image-derived shoot phenotypic traits (**Figures 7**, **8**). To cope with drought, plants induce protective mechanisms against water deficit. In addition to stomatal closure, assimilates are often re-allocated from the shoot to the root, thereby enhancing root growth and extension into deeper soil layers (Rich and Watt, 2013; Xu et al., 2015). Roots have the ability to plasticly change their spatial distribution in the soil in response to drought stress (Orman-Ligeza et al., 2018; Orosa-Puente et al., 2018; von Wangenheim et al., 2020). The degree of plasticity, however, is trait and genotype dependent. As mentioned by Tracy et al. (2020), phenotypes in roots and shoots are expressed differently depending on environmental conditions. Our results show that roots respond faster to drought than shoots as significant differences between drought and WW treatments occurred 10 days after drought imposition in roots, while in shoots significant differences could only be observed 4 days later (**Table 2**). Drought affected not only root biomass, which was represented by TRV, but also modified the morphology of root with changes in other traits, such as TRL, and also the root diameter.

Notably, there was a tendency for TRL to increase shortly after the onset of drought stress, although the difference was not significant. Sharp and Davies (1979) showed that a mild degree of water stress resulted in a higher root elongation rate compared to well-watered maize. The rate of root growth probably depends on the degree and the duration of the stress. Under extreme water deficit, root growth will be inhibited in many species (Rich and Watt, 2013; Kou et al., 2022). It is therefore highly important to assess the dynamics of root growth changes over time and under different conditions to gain deeper insights into the responsible mechanisms. Addressing only single time points will be inappropriate. Our high-throughput phenotyping platforms supports such measurements and thus the elucidation of the responses of plant organs to environmental cues and the adaptation to stress conditions.

The biomass ratio between well-watered and drought-treated plants provides a parameter to compare different genotypes with respect to their response to drought (Harb et al., 2010; Correia et al., 2022). Among the five inbred lines tested here, B73 exhibited the lowest ESV ratio at most time points. This suggests that B73 was most severely affected by drought, which is in line with the study of Chen et al. (2012), who classified B73 as a drought-sensitive line. The estimated TRL and TRV ratios suggested that S052 produced more roots, likely through enhanced lateral root growth and this effect lasted also longer than in the other lines. This might partly result from the relatively small shoot biomass (and transpiration) and the lower water consumption of the plants of this line (**Figure 10**), which caused a longer time period to reach water deficit than for other genotypes.

In future, some features of the phenotyping installation need to be taken into account when it is used to assess genotypic difference in responses to drought: due to the relatively large volume of the rhizo-pots compared to regular growth pots, soil moisture levels will decrease more slowly. This is closer to the natural drought scenarios in the field, where gradual changes in water availability occur rather than abrupt changes (Poorter et al., 2012). Soil drying in the rhizo-pots occurs due to the water consumption by the plants and the evaporation from the soil surface. While the latter is rather uniform (also due to the use of the soil cover), the former is affected by the size of the plants and by their physiological states, in particular their rates of transpiration. The impact of small plant size was evident for the line N22, which did not suffer from the drought stress as much as the other lines, since the FC was still 40% at the end of the experiment (**Supplementary Figure 4**). This is probably the main reason why the reduction of shoot mass was least compared to the other genotypes. A similar tendency could be observed for many other measured traits. Also, most colour-related traits did not differ after water deprivation, which might also be caused by the weak stress imposed in the system. If the different water consumption of plant lines under investigation (mainly due to different plant sizes) cannot be avoided by an appropriate selection of the population under investigation, we suggest adjusting the drought regime imposed to all plants to that of the plants with the lowest water consumption. This could be achieved by programming a gradual decrease of the watering target weight, which is gauged to the weakest water consumer, rather than a complete stop of watering. To avoid a too long period to reach considerable water stress levels, the soil moisture level in the rhizo-pot used in the initial phase of the experiment should be carefully adjusted.

Plant productivity is the results of integrating processes occurring in both the root and the shoot systems. Therefore, a deeper understanding of the effects of different stresses such as water deficit or nutrient limitation on roots and shoots is of great value for cultivar selection, the improvement of crop models, and low-input agriculture (Hadir et al., 2020). Our observations confirm that the root phenotyping upgrade of the platform supports experiments to assess the dynamic phenotypic changes of both shoots and roots (and thus the relations of these two important organ systems) caused by genetic variation and/or induced by environmental triggers such as drought stress.

## Conclusion

With the latest updates, the IPK automated high-throughput phenotyping platform for large plants is capable of capturing images from both shoots and roots. Consequently, the dynamic growth patterns among various genotypes in up to 396 carriers/plants, as well as the response to different environmental scenarios, can be analysed in a single experiment.

This study illustrates the applicability and importance of this system. Combing growth-related shoot and root traits helps us to better interpret the difference between hybrid and inbred lines. Moreover, it sheds some light on the hidden parts of plants and illustrated the early response of roots to drought. Genotypic differences in adaptation were identified in the five inbred lines. The assessment of dynamic growth from more diverse lines with different degrees of drought resilience will be helpful to explore the underlying mechanisms and to obtain more information about the shoot–root relationships in response to drought. The integrated shoot and root phenotyping platform can also be applied to investigate other stress responses or nutrient deficiency scenarios for large plant species.

## Data availability statement

The raw data supporting the conclusions of this article will be made available by the authors, without undue reservation.

## Author contributions

TA and AJ designed the experiment. RS and CS performed the experiment. RS and DK analysed the data. RS drafted the manuscript with input from CS, DK, and TA. TA and AJ supervised the project. All authors contributed to the article and approved the submitted version.
