# Supplementary material for: Integrated phenotyping of root and shoot growth dynamics in maize reveals specific interaction patterns in inbreds and hybrids and in response to drought
Source: Front Plant Sci. 2023 Sep 1;14:1233553. doi: 10.3389/fpls.2023.1233553 (PMC10502302; doi:10.3389/fpls.2023.1233553)
Supplement: Supplementary file 1 [file DataSheet_1.pdf]

Supplementary material

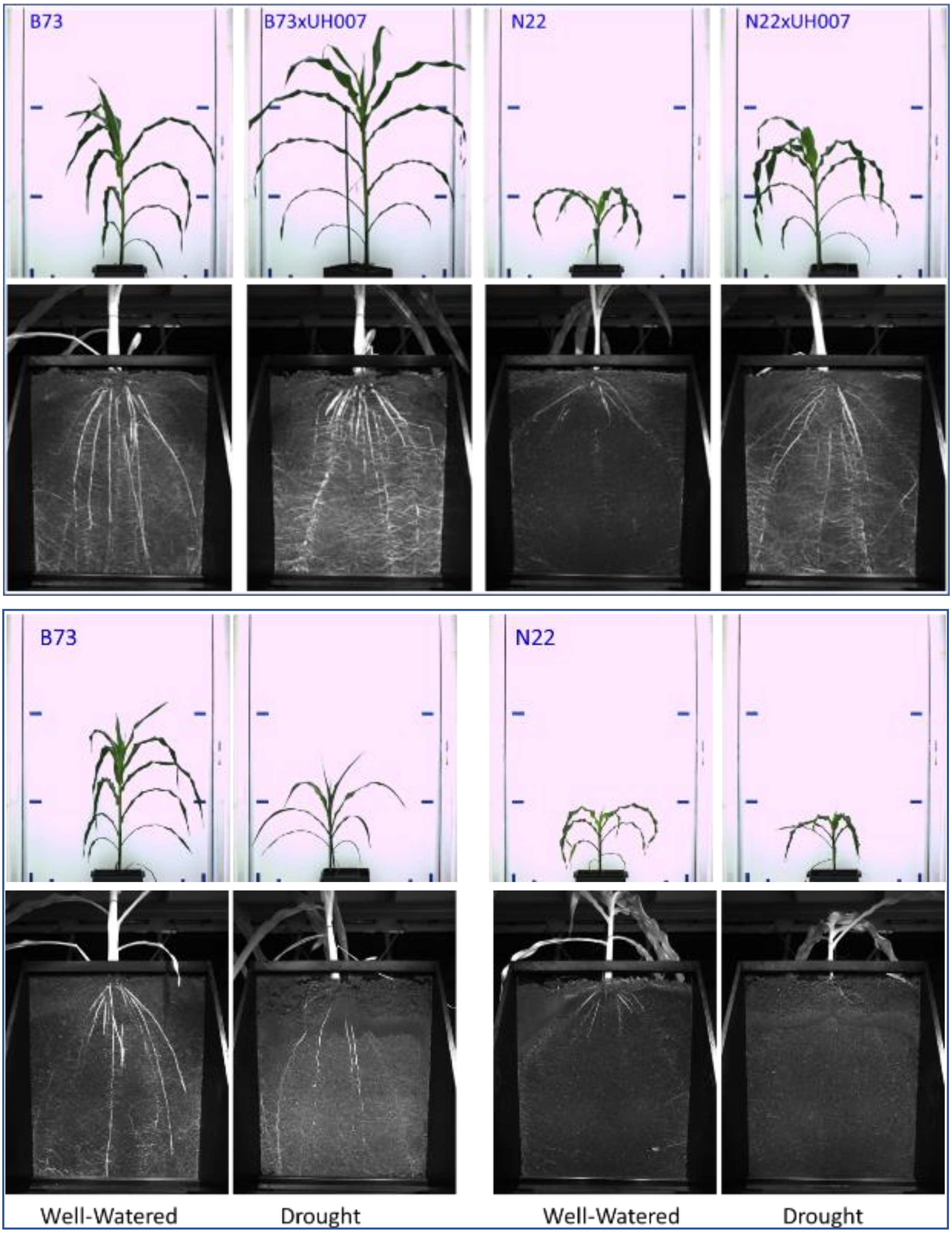

Supplementary Figure 1. Shoot and root phenotypes of the maize inbred lines B73 and N22 and the hybrids B73xUH007, N22xUH007 under well-watered and drought conditions at the end of the experiment at 40 DAT (days after transplanting). The shoot images were taken from the side view by an RGB camera and the root images were taken by a NIR camera in

the automated high-throughput phenotyping platform. The plants were grown in a climatized greenhouse. The drought stress was initiated at 13 DAT and the field capacity (FC) was then kept at 35%.

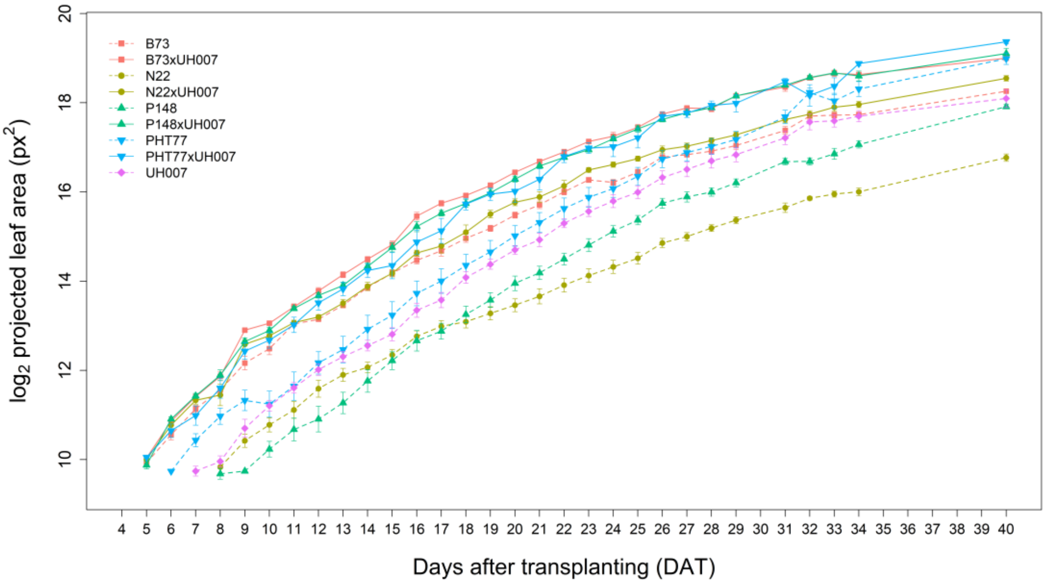

Supplementary Figure 2. Projected leaf area derived from the images of hybrid and inbred maize plants over time. Data are shown as means of nine replicates and the error bars denote ±SE.

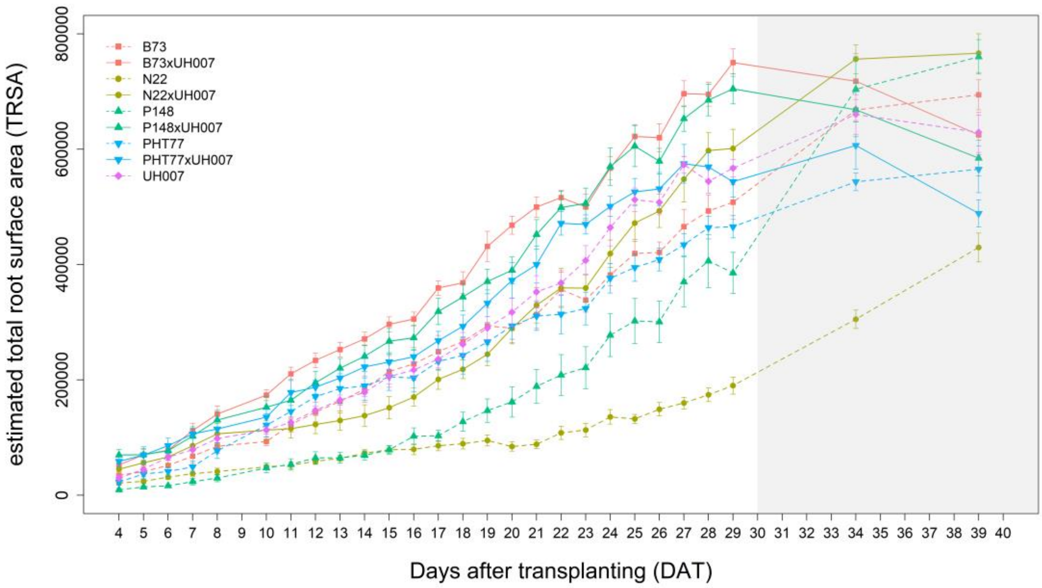

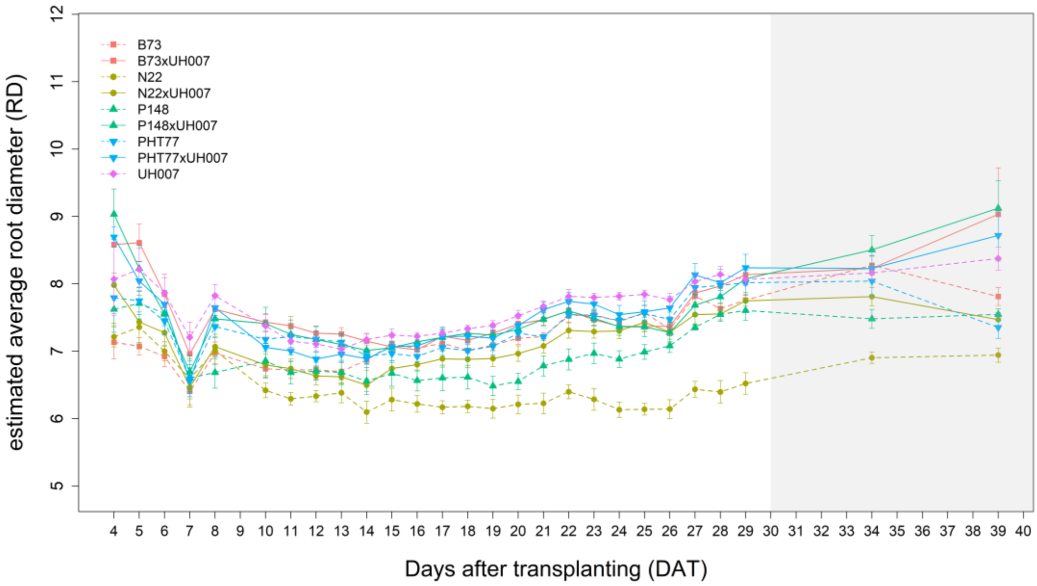

Supplementary Figure 3. Estimated total root surface area (TRSA) and average root diameter (RD) extracted by saRIA (semi-automated Root Image Analysis) software from the NIR-images of hybrid and inbred maize plants over time. Data are shown as means of nine replicates and the error bars denote ±SE. The grey area marks time points with data of low reliability due to the increasing density of roots and their progressive merging and overlapping. Values derived from images taken at 34 and 39 DAT are included only for illustration.

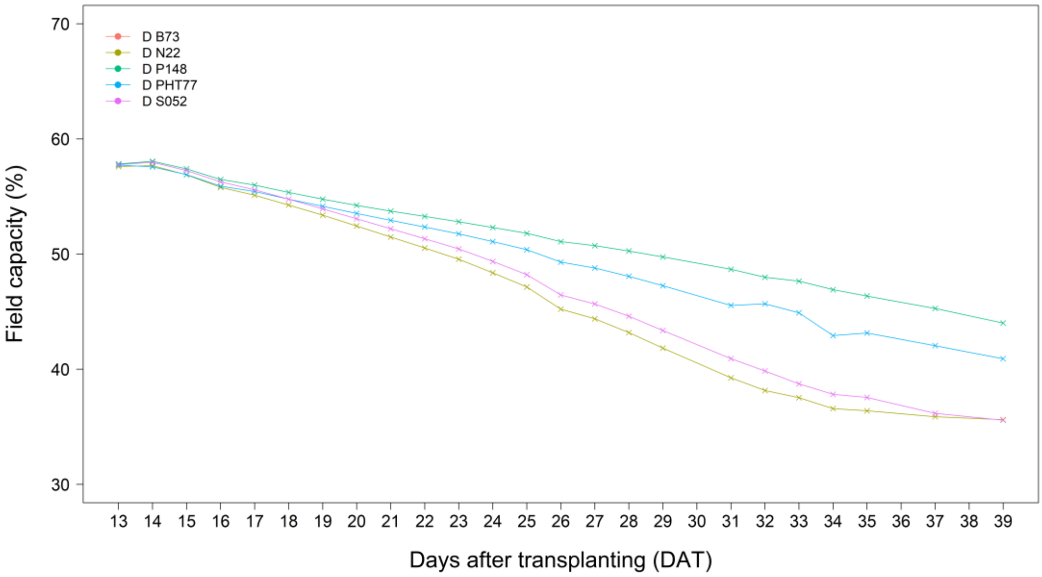

Supplementary Figure 4. Field capacity (FC, %) calculated according to the weight of the pots for the five inbred lines under drought condition, which was initiated at 13 DAT. The FC was kept at 35% for drought (D) and 60% for well-watered (WW) treatment. The values denote the mean of each line (n=9) over time.

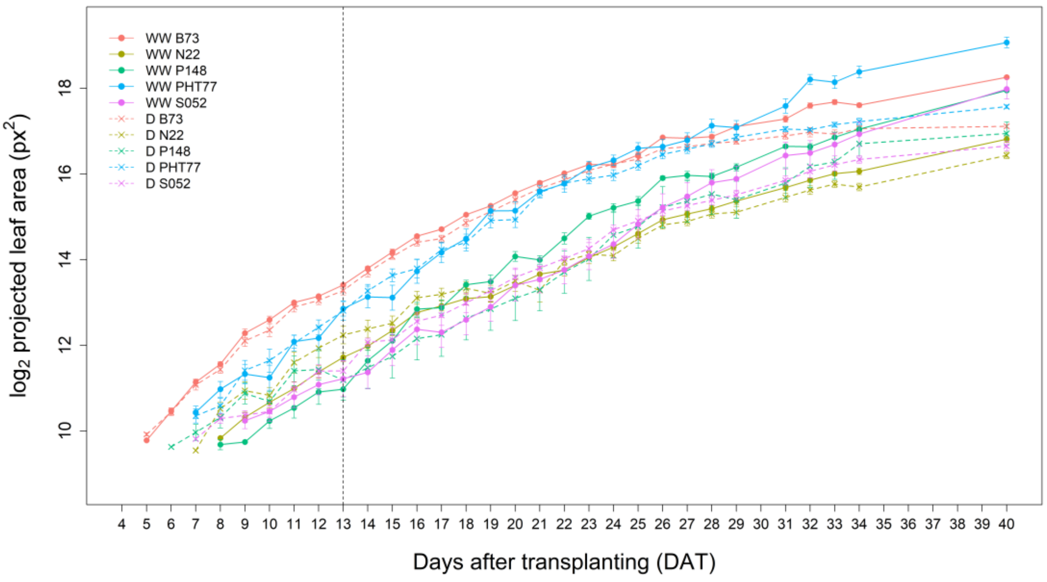

Supplementary Figure 5. Projected leaf area derived from the images of well-watered (WW) and drought-treated (D) plants over time. Data are shown as means of nine replications and the error bars denote ±SE. The vertical dashed line denotes the starting time of imposing drought stress at 13 DAT.

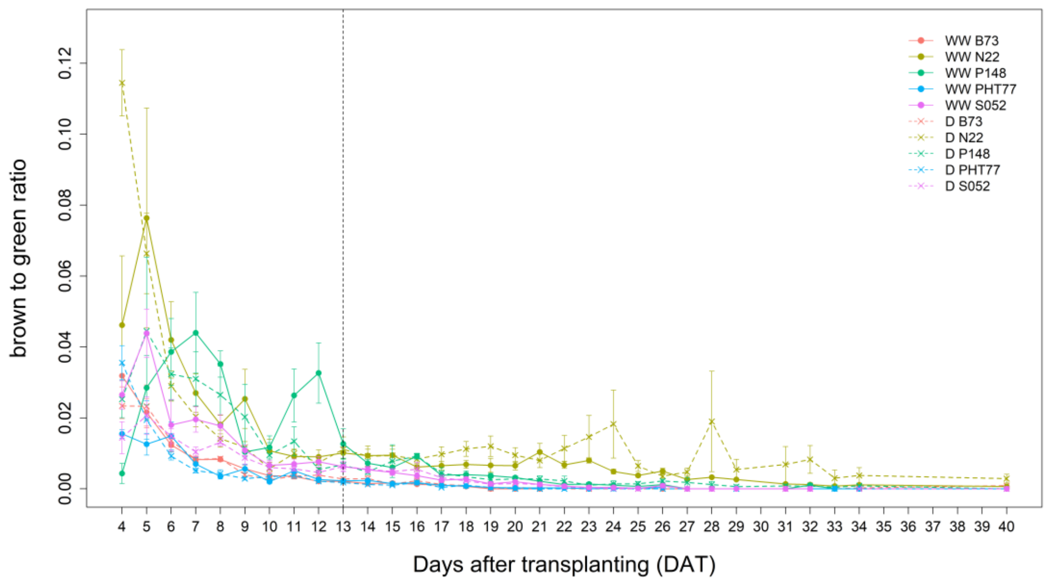

Supplementary Figure 6. Color-related phenotyping trait, brown to green derived from the images of well-watered (WW) and drought-treated (D) plants over time. Data are shown as means of nine replications and the error bars denote ±SE. The vertical dashed line denotes the starting time of imposing drought stress at 13 DAT.

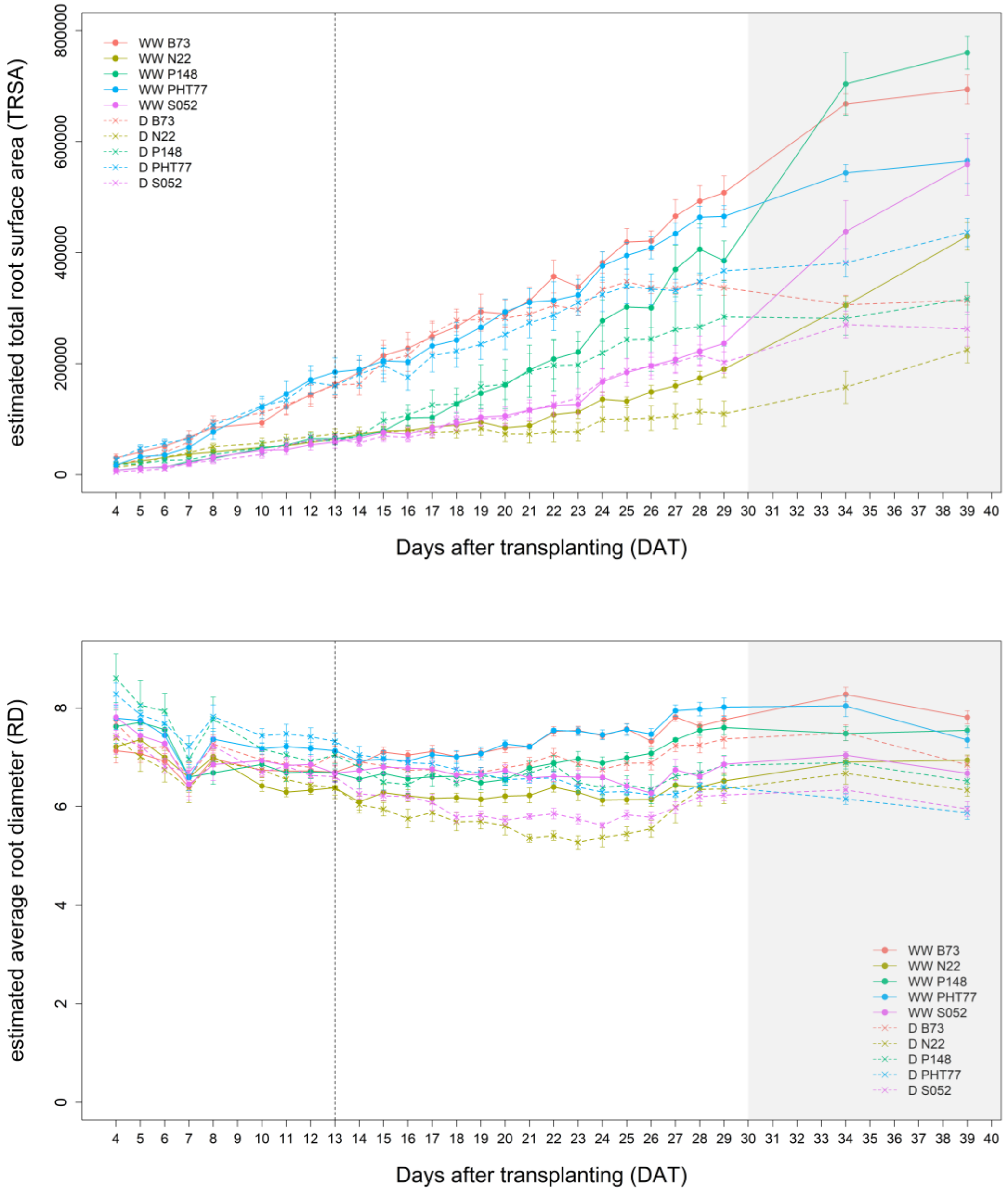

Supplementary Figure 7. Root phenotyping traits, total root surface area (TRSA) and average root diameter (RD) extracted by saRIA (semi-automated Root Image Analysis) software from the NIR-images of well-watered (WW) and drought-treated (D) plants over time. Data are shown as means of nine replications and the error bars denote ±SE. The vertical dashed line denotes the starting time of imposing drought stress at 13 DAT. The grey area marks time points with data of low reliability due to the increasing density of roots and their progressive merging and overlapping (in particular in the WW plants). Values derived from images taken at 34 and 39 DAT are included only for illustration.
